# Molecular Targeted Positron Emission Tomography Imaging and Radionuclide Therapy of Pancreatic Ductal Adenocarcinoma

**DOI:** 10.3390/cancers13246164

**Published:** 2021-12-07

**Authors:** Thomas T. Poels, Floris A. Vuijk, Lioe-Fee de Geus-Oei, Alexander L. Vahrmeijer, Daniela E. Oprea-Lager, Rutger-Jan Swijnenburg

**Affiliations:** 1 Department of Surgery, Cancer Center Amsterdam, Amsterdam UMC, Vrije Universiteit Amsterdam, De Boelelaan 1117, 1081 HV Amsterdam, The Netherlands; r.j.swijnenburg@amsterdamumc.nl; 2 Department of Surgery, Leiden University Medical Center, Albinusdreef 2, 2333 ZA Leiden, The Netherlands; f.a.vuijk@lumc.nl (F.A.V.); a.l.vahrmeijer@lumc.nl (A.L.V.); 3 Department of Radiology, Leiden University Medical Center, Albinusdreef 2, 2333 ZA Leiden, The Netherlands; l.f.de_geus-oei@lumc.nl; 4 Department of Radiology and Nuclear Medicine, Cancer Center Amsterdam, Amsterdam UMC, Vrije Universiteit Amsterdam, De Boelelaan 1117, 1081 HV Amsterdam, The Netherlands; d.oprea-lager@amsterdamumc.nl

**Keywords:** pancreatic ductal adenocarcinoma, positron emission tomography, radionuclide, tumour tracer

## Abstract

**Simple Summary:**

Pancreatic ductal adenocarcinoma (PDAC) has a dismal prognosis, mainly due to difficulty in early detection of the disease by current imaging modalities. In this review, we discuss the more specific diagnostic imaging modality that evaluates the presence of specific tumour tracers via positron emission tomography. In addition, we review the available therapeutic applications of these tumour-specific tracers.

**Abstract:**

Pancreatic ductal adenocarcinoma (PDAC) has an inauspicious prognosis, mainly due to difficulty in early detection of the disease by the current imaging modalities. The upcoming development of tumour-specific tracers provides an alternative solution for more accurate diagnostic imaging techniques for staging and therapy response monitoring. The future goal to strive for, in a patient with PDAC, should definitely be first to receive a diagnostic dose of an antibody labelled with a radionuclide and to subsequently receive a therapeutic dose of the same labelled antibody with curative intent. In the first part of this paper, we summarise the available evidence on tumour-targeted diagnostic tracers for molecular positron emission tomography (PET) imaging that have been tested in humans, together with their clinical indications. Tracers such as radiolabelled prostate-specific membrane antigen (PSMA)—in particular, ^18^F-labelled PSMA—already validated and successfully implemented in clinical practice for prostate cancer, also seem promising for PDAC. In the second part, we discuss the theranostic applications of these tumour-specific tracers. Although targeted radionuclide therapy is still in its infancy, lessons can already be learned from early publications focusing on dose fractioning and adding a radiosensitiser, such as gemcitabine.

## 1. Introduction

Pancreatic ductal adenocarcinoma (PDAC) is the most frequent type of all pancreatic cancers and has an inauspicious prognosis, with a five-year survival rate of less than 5% [1,2]. This extremely low survival rate is mainly due to difficulty in early detection of the disease by the current imaging modalities. Staging and, hence, rational use of treatment are highly dependent on information yielded from conventional imaging modalities (i.e., computed tomography (CT), magnetic resonance imaging (MRI), or endoscopic ultrasound (EUS) [3]. However, almost 50% of surgeries are performed without patient benefit (i.e., due to benign diagnoses, undetected metastases, or rapid recurrence <6 months), indicating that these imaging modalities are lacking diagnostic precision and therapy response evaluation accuracy.

During surgery for PDAC, 10% of the patients already present with metastases at laparoscopy, and approximately half of the patients undergoing a resection will have microscopically positive resection margins (R1), of whom 25% will develop disease recurrence within six months after surgery. Furthermore, the imaging in patients with borderline resectable or locally advanced PDAC who started chemotherapy is unreliable due to the difficulty in distinguishing between fibrosis and stroma in PDAC [4]. Additionally, ^18^F-fluorodeoxyglucose (^18^F-FDG) PET-CT, the most commonly used tracer in oncology, has a variable and debatable role in the routine pancreatic work up, mainly due to the large number of false positive findings by also identifying pancreatitis, potentially resulting in futile resections of the pancreas. ^18^F-FDG PET-CT is therefore only reserved on indication for the individual patient [4].

The upcoming development of tumour-specific tracers provides an alternative solution for more accurate diagnostic techniques, staging, and therapy response monitoring. Targeted radionuclides such as radiolabelled peptides, which bind to the receptors overexpressed by cancer cells, and radiolabelled antibodies to tumour-specific antigens can provide a more specific diagnosis [5,6,7]. Additionally, this development offers new possibilities to maximally capitalise on the theranostic applicability, i.e., the possibility to use the tracer both for imaging purposes as well as a targeting binder for radionuclide therapy.

In the first part of this review, we summarise the available evidence on tumour-targeted imaging tracers for molecular PET-CT imaging that have been tested on humans, together with their clinical indications, and in the second part, we discuss the theranostic applications of these tumour-specific tracers.

For this narrative review, our search strategy for both the diagnostic and therapeutic sections consisted of a general search of diagnostic and therapeutic tracers in pancreatic cancer, followed by a search of specific tracers and, finally, reviewing the papers for leads to other—not yet included—tracers.

## 2. Part 1: Tumour-Targeted Tracers for the Detection of Pancreatic Cancer

Early detection is important for the treatment of PDAC. It is believed there are two main precursors for PDAC, namely pancreatic intraepithelial neoplasia (PanIN) and intraductal papillary mucinous neoplasm (IPMN).

Three grades can be distinguished in PanIN. PanIN-1 and PanIN-2 are commonly found in patients over the age of 40 or in chronic pancreatitis. PanIN-3 is more exclusively found in the pancreas with PDAC and is the stage prior to invasion [8,9,10].

IPMNs develop in the cells lining the pancreatic ducts and contribute to mucin production, cystic dilatation of the pancreatic ducts, and intraductal papillary growth. IPMNs are at risk of developing into malignancy 20% of the time over a period of 10 years [8,11,12].

PanINs are challenging to diagnose, as all types are under the resolution of conventional imaging, due to their limited size. EUS may help in detecting identifiable parenchymal changes, such as acinar cell loss, the proliferation of small ductular structures, and fibrosis. This combination of changes, labelled as lobulocentric atrophy (LCA), however, are not specific to PanIN [8,13].

In diagnosing PDAC, an important challenge is to distinguish PDAC from pancreatitis, as both entities have abundant stroma. Additionally, neoadjuvant treatment such as FOLFIRINOX makes it difficult to discriminate between viable tumour and chemoradiation-induced tumour necrosis and fibrosis [14,15,16].

Tumour-targeted molecular imaging could provide essential knowledge in these situations by adding metabolic molecular imaging information to the anatomical changes.

### 2.1. ^18^F-Fluorodeoxyglucose—^18^F-FDG

**Figure 1:**
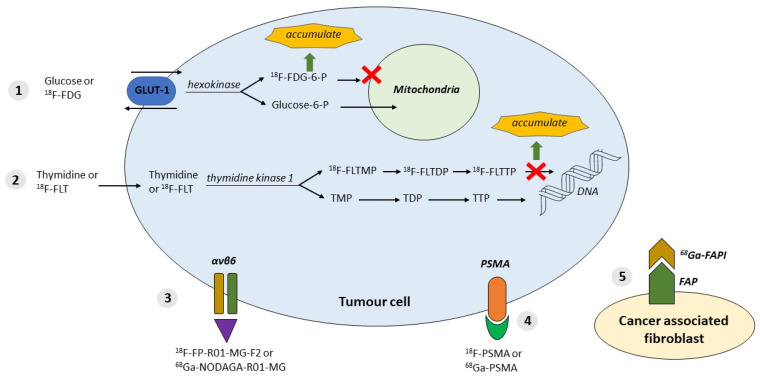
Available tracers and their properties. **1. ^18^F-FDG** tumour cells with the property of using glucose; GLUT-1 and hexokinase are upregulated in tumours. ^18^F-labelled FDG accumulation. **2. ^18^F-FLT**: cell proliferation in tumour cells; thymidine kinase is upregulated. ^18^F-labelled (FLT)→FLTTP accumulation. **3. *αvβ6*** overexpressed in tumour cells; targeted by labelled peptides: **^18^F-FP-R01-MG-F2** or **^68^Ga-NODAGA-R01-MG**. **4. PSMA** expressed in tumour cells; targeted by inhibitor molecules labelled with ^18^F or ^68^Ga. **5.** Expression of **FAP** (fibroblast activation protein) by CAF (cancer-associated fibroblasts) targeted by FAPI (FAP inhibitor) labelled with ^68^Ga or ^18^F. Abbreviations: αvβ6 = integrin αvβ6; DNA = deoxyribonucleic acid; FAP= fibroblast activation protein; ^18^F = ^18^Fluorodeoxyglucose-labelled; ^18^F-FDG = ^18^F-fluorodeoxyglucose; ^18^F-FDG-6-P = ^18^F-FDG -6-phosphate; ^18^F-FLT = ^18^F-fluorothymidine; ^18^F-FLTDP = ^18^F-FLT diphosphate; ^18^F-FLTMP = ^18^F-FLT monophosphate; ^18^F-FLTTP = ^18^F-FLT triphosphate; ^18^F-FP-R01-MG-F2 = ^18^F-labelled integrin tracer; ^18^F-PSMA = ^18^F-labelled PSMA; ^68^Ga= ^68^Gallium-labelled; ^68^Ga-FAPI = ^68^Ga-labelled fibroblast activation protein inhibitor; ^68^Ga-NODAGA-R01-MG = ^68^Ga-labelled integrin tracer; ^68^Ga-PSMA= ^68^Ga-labelled PSMA; Glucose-6-P = Glucose-6-Phosphate; GLUT-1 = glucose transporter type 1; PSMA= prostate-specific membrane antigen; TDP = thymidine diphosphate; TMP = thymidine monophosphate; TTP = thymidine triphosphate.

PET imaging for the diagnosis of PDAC uses ^18^F-FDG, a radiolabelled glucose [17]. ^18^F-FDG PET imaging relies on the property that a normal pancreas tissue has low glucose usage compared to PDAC. In PDAC, a KRAS mutation induces an overexpression of hexokinase-2 and the glucose cell membrane transporter, GLUT-1 [18]. ^18^F-FDG is accumulated by PDAC, where it is phosphorylated and consequently goes into metabolic arrest [17,19] (Figure 1 and Table 1).

The average sensitivity and specificity for detecting PDAC by ^18^F-FDG is reported to be superior to CT, with a sensitivity of 94% and specificity of 90% for ^18^F-FDG compared to 82% and 75%, respectively, for CT [14,18,20].

A major limitation of PET imaging with ^18^F-FDG is that glucose uptake can also be seen in inflammation, leading to similar appearances of pancreatitis and PDAC [14,21]. However, when the diagnosis of PDAC is correct, the degree of ^18^F-FDG uptake can predict tumour aggressiveness and survival [22,23].

In clinical practice, differentiation between pancreatitis and PDAC is possible by performing a dual-phase PET scan. This method consists of performing a PET scan at two different time intervals after the injection of the tracer. Pancreatic masses on PET images in pancreatitis have lower standardised uptake values (SUV), which further decrease in the delayed phase. However, there can be overlap in SUV values between inflammation and PDAC. Furthermore, dual-phase ^18^F-FDG PET imaging is very time-consuming and, therefore, not often feasible in daily practice [17,24].

The specificity of PET imaging for the diagnosis of PDAC could be improved by using more disease-specific imaging agents compared to ^18^F-FDG. Several other radiotracers have been used for the evaluation of PDAC [17,25,26]. These include radiotracers such as ^18^F-Fluorothymidine (^18^F-FLT), ^68^Gallium-labelled fibroblast activation protein inhibitor (^68^Ga-FAPI), ^68^Ga-labelled 1,4,7,10-tetraazacyclododecane-1,4,7,10-tetraacetic acid-FAPI-04 (^68^Ga-DOTA-FAPI-04), ^18^Fluorodeoxyglucose-labelled PSMA (^18^F-PSMA), ^68^Ga-labelled PSMA (^68^Ga-PSMA), and Integrin αvβ6 tracers. These tracers are amply discussed in the following sections.

### 2.2. ^18^F-Fluorothymidine—^18^F-FLT

^18^F-fluorothymidine is a marker of cell proliferation due to tracer accumulation in proliferating cells. Thymidine kinase activity is upregulated during proliferation, subsequently phosphorylating ^18^F-FLT, which gets trapped intracellularly (Figure 1 and Table 1).

^18^F-FLT PET imaging has shown a good correlation with histological Ki-67 expression, a marker of cell proliferation [17,27]. Furthermore, ^18^F-FLT PET imaging is potentially superior to ^18^F-FDG PET, as ^18^F-FLT uptake is not affected by inflammation or hyperglycaemia [17].

### 2.3. Fibroblast Activation Protein Inhibitor—FAPI

In PDAC, more than 90% of the tumour volume consists of cancer-associated fibroblasts (CAF). CAFs are associated with the promotion of tumour growth, tissue invasion, metastasis developing, and therapy resistance [28,29,30,31]. CAFs express Fibroblast Activation Proteins (FAP) on the cell surface, a type II membrane-bound glycoprotein [32,33]. FAP can be detected by performing a PET-CT with ^68^Ga-labelled FAP inhibitors (Figure 1 and Table 1).

Röhrich et al. showed in a small study including 19 PDAC patients (seven primary and 12 progressive/recurrent) that ^68^Ga-FAPI PET-CT led to restaging in half of the patients with PDAC and, also, in most patients with recurrent disease compared to standard of care imaging. Differentiation from pancreatitis was challenging but significantly improved with imaging at multiple time points after the injection of ^68^Ga-FAPI [28].

Chen et al. compared the use of ^68^Ga-DOTA-FAPI-04 to ^18^F-FDG PET for the diagnosis of the primary disease and metastatic lesions for various types of cancer. Four patients with pancreatic cancer were included. In one patient, pancreatic cancer was not visualised due to uptake throughout the pancreas caused by tumour-induced pancreatitis [34]. Identical findings of uptake of ^68^Ga-DOTA-FAPI-04 in patients with Ig-G4-related disease have been reported by others [34,35,36,37,38].

The study of Chen et al. did show a significantly lower uptake of ^68^Ga-DOTA-FAPI-04 than ^18^F-FDG, thus facilitating an improved detection of possible liver metastases [34].

### 2.4. Integrin αvβ6

Integrins are proteins that facilitate the adhesion of cells to the extracellular matrix (ECM) of polypeptides. Integrins play a crucial role in the signalling pathway for the regulation of cell differentiation, migration, proliferation, and apoptosis [39,40]. In many cancers, the expression of specific integrins can become dysregulated, such as αvβ3 and αvβ6. The overexpression of αvβ3 results in overpromotion of the angiogenesis pathway [39,41].

Integrin αvβ6 promotes PDAC by modulating the proliferation, survival, migration, and invasion of both the cancer cells and its microenvironment [42]. Studies have shown higher expressions of αvβ6 in PDAC compared to other type of cancers [39,43,44]; additionally, the differentiation of PDAC from pancreatitis was possible [45]. Tumour-positive lymph nodes also showed elevated levels of αvβ6 [45].

As αvβ6 seems to be an important integrin for the detection of PDAC and distinguished from pancreatitis, numerous research groups have been developing PET tracers [46,47,48].

Kimura et al. used ^18^F-FPR01-MG-F2 to target αvβ6 (Figure 1 and Table 1). The study group demonstrated that the targeting peptide was able to penetrate the pancreatic tumour rapidly and also showed an improved uptake compared to ^18^F-FDG, reflecting the difference in the peptide’s target, namely glucose metabolism versus expression of the ECM protein. In addition, it was observed that uptake only occurred in the viable part of the tumour compared to the parts with significant necrosis [39].

A recent small study from Quigley et al. showed the first promising results for ^68^Ga-labelled trimerized αvβ6-integrin-selective nonapeptide (^68^Ga-Trivehexin)-enabled PET-CT imaging. One patient, out of a total of four, was included with PDAC, showing a high tracer uptake in the pancreatic tumour, including multiple liver metastases [49].

### 2.5. Prostate-Specific Membrane Antigen Targeted PET-CT Imaging

Currently, different types of radiolabelled PSMA tracers exist (i.e., ^18^F-PSMA and ^68^Ga-PSMA) with different biodistributions, as largely described in the literature [50].

Prostate-specific membrane antigen (PSMA) is a type II transmembrane glycoprotein highly expressed on the surfaces of prostate cancer cells. The expression of PSMA in the tumour-associated (neo)vasculature of prostate cancer, breast cancer and primary gliomas has been reported and has also been proven to be high in PDAC [51,52,53].

The immunohistochemical experiments from our group showed high expression of PSMA in four out of five patients with PDAC de novo, as well as in 32 out of 33 PDAC patients after neoadjuvant treatment (mean tumour H-score of 99 (maximum 300)). These experiments also showed no expression in adjacent normal and pancreatitis tissue (H-score 0), thus yielding a high tumour contrast with the background and improved tumour detection [54].

Radiolabelled PSMA-targeted PET-CT has proven highly successful for the primary staging and restaging of prostate cancer patients and is currently being implemented worldwide [55,56]. PSMA expression can be imaged by labelling small inhibitor molecules with PET radionuclides, i.e., ^18^F or ^68^Ga [57] (Figure 1 and Table 1).

### 2.6. ^18^F-labelled Prostate-Specific Membrane Antigen—^18^F–PSMA

^18^F-radiolabelled PSMA PET has been recently technically validated and successfully implemented in clinical practice for prostate cancer [54,55,58].

One of the more commonly used variants, ^18^F-DCFPyL, a second-generation ^18^F-fluorinated PSMA ligand, has advantages over ^68^Ga-labelled PSMA tracers. It provides a higher spatial resolution, along with a longer half-life, which may result in more accurate staging due to the detection of small local tumour deposits [59].

### 2.7. ^68^Ga-labelled Prostate-specific Membrane Antigen—^68^Ga-PSMA

Krishnaraju et al. showed improved diagnostic accuracy with ^68^Ga-PSMA compared to ^18^F-FDG in a study among 40 patients with pancreatic lesions—positive predictive value 90.5% versus 65.4% for ^68^Ga-PSMA compared to ^18^F-FDG; accuracy 92.5% versus 72.5%, respectively [57].

## 3. Part 2: Targeted Radionuclide Therapy of Pancreatic Ductal Adenocarcinoma

Locally advanced and metastatic pancreatic cancer have poor prognoses. The current standard of care treatments such as gemcitabine or FOLFIRINOX provide minimal survival benefits. Targeted radionuclide therapy may provide improved survival in addition to less systemic toxicity than seen with the current chemotherapy [60].

In patients with cancer, the ultimate goal is, first, to identify the receptor expression (by using a diagnostic scan with a diagnostic tracer) and, then, in the case of an adequate expression of the receptor, to use the tracer, radiolabelled with alpha or beta-particles, for therapeutic purposes, with curative or sometimes palliative intent [61].

In addition to therapy with radiolabelled antibodies, there is a possibility to add gemcitabine, serving as a radiosensitiser, which is generally well-tolerated in combination with external radiotherapy [62,63,64,65,66].

In this second part, we will discuss the theranostic applications of tumour-specific tracers. To our knowledge, there have only been two different types of antibodies that have been reported on for humans as a targeted radionuclide therapy of PDAC: ^131^I-KAb201 antibody and ^90^Y-clivayuzumab tetraxetan antibody (^90^Y-labelled hPAM4). Furthermore, there is only one single study registered at the popular databases that is currently recruiting patients and focusing on the theranostic pair of ^68^Ga-DOTA-5G /^177^Lu-DOTA-ABM-5G [67].

### 3.1. ^131^I-labelled KAb201 Antibody—^131^I-KAb201

Carcinoembryonic antigen (CEA) is expressed in most patients with pancreatic cancer and therefore serves as an interesting target for antibodies. One potential antibody is KAb201, an anti-CEA antibody labelled with ^131^Iodine [68].

Sultana et al. performed a randomised phase I/II trial assessing the safety and efficacy of ^131^I-KAb201 in patients with inoperable PDAC. Patients were randomised to receive ^131^I-KAb201 via either the intra-arterial (gastroduodenal artery) or intravenous (standard intravenous line) delivery route. The hypothesis of including an intra-arterial delivery route is expected to have a higher concentration of the radiolabelled drug at the target site and, thus, increased effectiveness with reduced toxicity [68].

In total, nineteen patients were randomised (nine in the intravenous arm, 10 in the intra-arterial arm), of whom one patient was excluded from the intra-arterial arm, as there was no uptake on the pretherapy scan. The overall response rate was 6% (one out of 18 patients). Dose-limiting toxicity was only reached in the intra-arterial route (at 50 mCi). Both anti-chimeric antibodies (HACA) and anti-sheep antibodies (HASA) were developed for the entire study population, thus limiting the possibility of repeat dosing, as this could lead to either hypersensitivity reactions or to complexing with circulating antibodies, creating a challenge to maintain effective therapeutic levels [68,69].

The median overall survival was 5.2 months (95% CI = 3.3–9.0 months), with no significant difference between either delivery arm (log-rank test *p* = 0.79) [70]. Survival and efficacy data were comparable with a single agent therapy of gemcitabine [71,72,73].

Future improvements can be found in the ability to predict the occurrence and type (I or II) of antibody response, thus aiding the possibility of repeat dosing. Additionally, humanisation of the antibody may reduce the immunogenicity [68].

### 3.2. ^90^Y-labelled Clivazutumab Tetraxetan Antibody—^90^Y-hPAM4

Preclinical studies in nude mice have shown that ^90^Y-labelled PAM4 decelerates tumour growth [70,74]. PAM4 is a monoclonal antibody that binds to a mucin produced primarily in PDAC [75,76,77,78].

Gulec et al. performed a phase I single-dose escalation trial among 21 patients with PDAC (four stage III—locally advanced; 17 stage IV—metastatic) with the primary aim of determining the dose-limiting toxicity and the maximum tolerated dose. Patients first received ^111^In-hPAM4 for diagnostic imaging and, finally, ^90^Y-hPAM4 for therapy [79].

The drug-related toxicities among the study group were grade 3/4 neutropenia and thrombocytopenia, which both increased with the ^90^Y dose. Fourteen patients progressed rapidly; however, seven patients remained progression-free for 2–6 months, with three patients showing a partial response with tumour shrinkage. The combination with gemcitabine showed further improvements [79].

Fractionated dosing of ^90^Y-hPAM4 (for an increased total radiation dose) in combination with gemcitabine acting as a radiosensitiser (for an increased potency of the radiation) could be a promising treatment regimen [38,79].

Ocean et al. showed in a phase 1 trial among 38 untreated patients with pancreatic cancer (five stage III—locally advanced, 33 stage IV—metastatic) that the fractionated dosing of ^90^Y-hPAM4 in combination with gemcitabine in repeated cycles (the number of cycles variated among the patients) allowed for double the radioimmunotherapy dose [80].

The drug-related toxicities among the study group were grade 3/4 thrombocytopenia and neutropenia in 28 patients. Sixteen patients showed stabilisation and six patients a partial response. The median overall survival was 7.7 months for all patients, with improved survival up to 11.8 months with repeated cycles [80].

An important terminated and unpublished study is the PANCRIT-1 trial. This was an international, multi-centre, double-blinded, randomised phase III trial of ^90^Y-labelled hPAM4 in combination with gemcitabine versus a placebo in combination with gemcitabine in patients with metastatic PDAC who had progressed despite receiving at least two prior therapies for metastatic disease. After the enrolment of 334 patients, an interim analysis on overall survival was performed, showing that the treatment arm did not demonstrate a sufficient improvement in the overall survival [81]. One major flaw of the study seemed to be the lack of a pre-treatment evaluation of the receptor expression, thus not applying the theranostic concept.

### 3.3. ^68^Ga-DOTA-5G /^177^Lu-DOTA-ABM-5G Theranostic Pair

(^68^Gallium-labelled—DOTA—G/^177^labelled Lutetium—DOTA–ABM-5G; DOTA = 1,4,7,10-tetraazacyclododecane-1,4,7,10-tetraacetic acid).

The only study listed in a clinical study database (ClinicalTrials.gov) is from Sutcliffe et al. from the University of California. This is a phase I study evaluating the safety and efficacy of the theranostic pair of ^68^Ga-DOTA-5G /^177^Lu-DOTA-ABM-5G in patients with locally advanced or metastatic PDAC. Patients will first receive a diagnostic ^68^Ga-DOTA-5G PET scan; subsequently, only the patients that show any uptake will receive ^177^Lu-DOTA-ABM-5G as a therapy.

The primary objective is to identify the dose limiting toxicity and the recommend phase 2 dose. The objective is to enrol 30 participants, with an expected completion in 2023 [67].

In summary, the results from the study with ^131^I-KAb201 demonstrated the importance of further investigation into the type of antibody response and the ability to predict this adverse event for the possibility of repeat dosing. Future research will learn if humanisation of the antibody is able to reduce immunogenicity. Additionally, the route of delivery (intra-arterial versus intravenous) did not show any difference in survival benefit or reduction in toxicity. Studies using ^90^Y-labelled hPAM4 proved that dose fractioning could be successful in increasing the total radiation dose without increasing the adverse events. In addition, a combination with gemcitabine acting as a radiosensitiser can increase the potency of the radiation. Future research, including randomised controlled trials, will need to confirm these results.

We are looking forward to the first results from the ^68^Ga-DOTA-5G/^177^Lu-DOTA-ABM-5G theranostic pair, as an evaluation of the quantifiable antibody localisation at the site of disease before administering a therapeutic dose seems to be the best tailormade medicine.

## 4. Summary and Challenges for the Future

Impressive efforts have been made in improving tumour-specific tracers for the detection of PDAC. In patients with PDAC, the ultimate goal is to firstly identify the radiolabelled peptide expression and, then, in the case of an adequate expression of the peptide, to use the tracer, radiolabelled with alpha or beta-particles, for therapeutic purposes, with curative or sometimes palliative intent.

18F-FDG is a well-known radiotracer that already is being used in PDAC. An important limitation, however, is that increased glucose metabolism is not specific for malignant processes only but can also be found in inflammatory and infectious disease sites. The specificity of PET imaging for the diagnosis of PDAC could be improved by a more disease-specific imaging agent.

**Figure 2:**
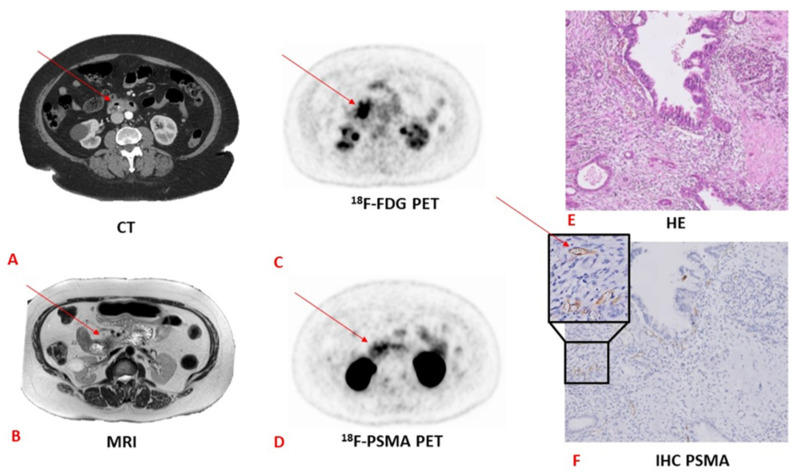
CT versus MRI versus ^18^F-FDG versus ^18^F-PSMA PET and the corresponding pathology and HE and immunohistochemistry tests in a patient with pancreatic adenocarcinoma. (**A**) CT image with an arrow pointing towards a pancreatic lesion. (**B**) MRI image with an arrow pointing towards a pancreatic lesion. (**C**) ^18^F-FDG PET image with an arrow pointing towards a pancreatic lesion. (**D**) ^18^F-PSMA PET image with an arrow pointing towards a pancreatic lesion. Note the more specific PSMA uptake compared to the ^18^F-FDG PET scan. (**E**) Haematoxylin and eosin staining (HE) image. Adenocarcinoma is not visible on this staining. (**F**) Immunohistochemistry staining of PSMA. The arrow points towards stained PSMA. CT, computed tomography; MRI, magnetic resonance imaging; PET, positron emitting tomography; HE, haematoxylin–eosin; IHC, immunohistochemistry. (The “HE” image has previously been published by our study group [54] and is licensed under a Creative Commons Attribution 4.0 International—http://creativecommons.org/licenses/by/4.0, accessed on 1 June 2021).

In general, there are currently three types of tracers: tracers that accumulate in the proliferating cell, such as ^18^F-FLT, and the second type of tracers target highly expressed integrin receptors or PSMA on the surfaces of cells in PDAC. In this group, ^18^F-PSMA is a promising tracer (Figure 2) that has already been validated and successfully implemented in clinical practice for prostate cancer. The last type of tracer targets fibroblast activation protein (FAP inhibitor), which is expressed by CAF (cancer-associated fibroblast).

Targeted radionuclide therapy is still in its infancy. The effectiveness of targeted radionuclide therapy has been limited by poor delivery to tumours. There have only been two different types of antibodies that have been reported on in humans as targeted radionuclide therapy of pancreatic ductal adenocarcinoma: ^131^I-KAb201 antibody and ^90^Y-clivayuzumab tetraxetan antibody (^90^Y-labelled hPAM4). Besides selecting the correct radionuclide antibody, the important contributing factors for successful therapy are dose fractioning and the addition of a radiosensitiser, such as gemcitabine.

These novel diagnostic and therapeutic approaches, in populations often characterised by poor outcomes and a decreased quality of life, have the potential to add a new chapter to patient’s lives.

## Figures and Tables

**Table 1:** Available tracers and their properties for the diagnosis of the primary disease.

Tracer	Properties	Localisation	Main Advantage	Main Disadvantage
^18^F-FDG	Marker of glucose consumption	Intracellular	High-glucose-use of malignant cells	High-glucose-using cells in inflammation
^18^F-FLT	Marker of cell proliferation	Intracellular	Cell proliferation in malignancies	
^68^Ga-FAPI; ^68^Ga-DOTA-FAPI-04	Expression of FAP by CAF targeted by FAPI labelled with ^68^Ga	Cell membrane of cancer-associated fibroblast	After multiple time points, PDAC and pancreatitis show a trend for differential uptake kinetics	Can be false positive in pancreatitis
^18^F-FP-R01-MG-F2; ^68^Ga-NODAGA-R01-MG; ^68^Ga-Trivehexin	Labelled peptides targeting *αvβ6* overexpressed in tumour cells	Cell membrane	Distinguishment between PDAC and pancreatitis. Additional uptake in lymph node metastases	
Radiolabelled PSMA (i.e.,^18^F-PSMA; ^68^GA-PSMA)	Inhibitor molecules labelled with ^18^F or ^68^Ga targeting PSMA expressed in tumour cells	Cell membrane	Very high diagnostic accuracy between PDAC and pancreatitis	

Abbreviations: αvβ6 = integrin αvβ6; CAF = cancer associated fibroblast; FAP = fibroblast activation protein; FAPI = FAP inhibitor; ^18^F = ^18^Fluorodeoxyglucose-labelled; ^18^F-FDG = ^18^F-fluorodeoxyglucose; ^18^F-FLT = ^18^F-fluorothymidine; ^18^F-FP-R01-MG-F2 = ^18^F-labelled integrin tracer; ^18^F-PSMA = ^18^F-labelled PSMA; ^68^Ga = ^68^Gallium-labelled; ^68^Ga-DOTA-FAPI-04 = ^68^Ga-labelled (macrocyclic chelator) 1,4,7,10-tetraazacyclododecane-1,4,7,10-tetraacetic acid-FAPI-04; ^68^Ga-FAPI = ^68^Ga-labelled fibroblast activation protein inhibitor; ^68^Ga-NODAGA-R01-MG = ^68^Ga-labelled integrin tracer; ^68^Ga-Trivehexin = ^68^Ga-labelled Trivehexin; ^68^Ga-PSMA = ^68^Ga-labelled PSMA; PSMA= prostate-specific membrane antigen.

## Abbreviations

αvβ6	integrin αvβ6
CT	computed tomography
CAF	cancer-associated fibroblast
CEA	carcinoembryonic antigen
DOTA	1,4,7,10-tetraazacyclododecane-1,4,7,10-tetraacetic acid
DNA	Deoxyribonucleic acid
ECM	extracellular matrix
EUS	endoscopic ultrasound
^18^F	^18^Fluorodeoxyglucose-labelled
FAP	fibroblast activation protein
FAPI	fibroblast activation protein inhibitor
^18^F-DCFPyL	^18^Fluorodeoxyglucose-labelled PSMA ligand
^18^F-FDG	^18^Fluorodeoxyglucose-labelled fluorodeoxyglucose
^18^F-FDG-6-P	^18^Fluorodeoxyglucose-labelled FDG -6-phosphate
^18^F-FLT	^18^Fluorodeoxyglucose-labelled fluorothymidine
^18^F-FLTDP	^18^Fluorodeoxyglucose-labelled FLT diphosphate
^18^F-FLTMP	^18^Fluorodeoxyglucose-labelled FLT monophosphate
^18^F-FLTTP	^18^Fluorodeoxyglucose-labelled FLT triphosphate
^18^F-FP-R01-MG-F2	^18^Fluorodeoxyglucose-labelled integrin tracer
^18^F-PSMA	^18^Fluorodeoxyglucose-labelled PSMA
^68^Ga	^68^Gallium-labelled
^68^Ga-FAPI	^68^Gallium-labelled fibroblast activation protein inhibitor
^68^Ga-DOTA-5G	^68^Gallium-labelled—DOTA—5G
^68^Ga-DOTA-FAPI-04	^68^Ga-labelled 1,4,7,10-tetraazacyclododecane-1,4,7,10-tetraacetic acid-FAPI-04
^68^Ga-NODAGA-R01-MG	^68^Ga-labelled integrin tracer
^68^Ga-PSMA	^68^Ga-labelled PSMA
^68^Ga-Trivehexin	^68^Gallium-labelled Trivehexin
Glucose-6-P	Glucose-6-Phosphate
GLUT-1	glucose transporter type 1
HACA	anti-chimeric antibodies
HASA	anti-sheep antibodies
HE	haematoxylin eosin
IHC	immunohistochemistry
^131^I-KAb201	^131^Iodine-labelled KAb201 antibody
IPMN	intraductal papillary mucinous neoplasm
KRAS	Kirsten rat sarcoma virus
LCA	lobulocentric atrophy
^177^Lu-DOTA-ABM-5G	^177^labelled Lutetium—DOTA–ABM-5G
MRI	magnetic resonance imaging
SUV	standardised uptake values
PanIN	pancreatic intraepithelial neoplasia
PDAC	pancreatic ductal adenocarcinoma
PET	positron emission tomography
PSMA	prostate-specific membrane antigen
TDP	thymidine diphosphate
TMP	thymidine monophosphate
TTP	thymidine triphosphate
^90^Y-hPAM4	^90^Yttrium-labelled clivazutumab tetraxetan antibody
